# Molecular surveillance of pneumococcal carriage following completion of immunization with the 13-valent pneumococcal conjugate vaccine administered in a 3 + 1 schedule

**DOI:** 10.1038/s41598-021-03720-y

**Published:** 2021-12-30

**Authors:** George A. Syrogiannopoulos, Ioanna N. Grivea, Maria Moriondo, Francesco Nieddu, Aspasia N. Michoula, Maria Rita Calabrese, Michael Anthracopoulos, Chiara Azzari

**Affiliations:** 1 Department of Pediatrics, University of Thessaly, School of Health Sciences, Faculty of Medicine, Biopolis, Larissa, Greece; 2 University of Florence and Anna Meyer Children’s Hospital, Florence, Italy; 3 Department of Pediatrics, University of Patras, Patras, Greece

**Keywords:** Epidemiology, Paediatric research, Molecular medicine

## Abstract

In a cross-sectional study, with the use of molecular methods, we aimed to gain insight into oropharyngeal pneumococcal colonization over time in 1212 Greek children recruited in general pediatric settings throughout the country; they were fully vaccinated with PCV13 (3 + 1 schedule). A single sample was obtained from each child at a time interval of 26 days to 70 months after administration of the 4th (booster) PCV13 dose; sampling time was divided into six time intervals. Carriage of *Streptococcus pneumoniae* was detected by real-time PCR targeting the *lytA* gene and isolates were serotyped by singleplex real-time PCR assays. Multiple control procedures to avoid false-positive results were applied. We showed an overall *S. pneumoniae* carriage rate of 48.6%. Serotyping identified typeable isolates in 82% of the total *lytA*-positive samples. Non-PCV13 serotypes represented 83.8% of total isolates when excluding serogroups with mixed PCV13 and non-PCV13 serotypes. In multivariate analysis daycare/school attendance emerged as the main contributing factor. Notably, serotypes 19A and 3 were the only two PCV13 serotypes the colonization rate of which increased over time (χ^2^ for trend *P* < 0.001 and *P* = 0.012, respectively). The application of the SP2020 gene on *lytA*-positive serotyped samples showed pneumococcal colonization in 97% of cases, and the overall colonization profile over time closely resembled that of the *lytA* gene. With the provisions of the methodological approach and age group of our study, the use of the oropharynx emerges as a reliable alternative to the nasopharynx in estimating pneumococcal carriage in epidemiological studies.

## Introduction

*Streptococcus pneumoniae* is a diverse pathogen which causes infections that range in severity from acute otitis media and sinusitis to pneumonia, septicemia, and meningitis^1–4^. The upper respiratory tract is the principal reservoir of *S. pneumoniae* in children and, nasopharyngeal sampling has been historically considered as the gold standard for the detection of *S*. *pneumoniae* carriage^4^. However, the organism also resides in the oropharynx^5–7^, and colonization of any part of the upper airway may lead to pneumococcal disease and transmission^4^. Therefore, it is not surprising that sampling of saliva in older children^5^ and the posterior oropharyngeal wall in adults^6–8^ has also been used as a site for pneumococcal detection.

Pneumococcal pharyngeal colonization and serotype characterization is routinely investigated by culture of the dominant isolate, followed by serotyping with specific antisera^9^. However, the use of traditional culture-based methods may miss serotypes present at a lower density^6,10^. This problem has been addressed by the recent development of molecular diagnostic methods, which offer high-sensitivity detection of multiple serotypes from a single naso-/oropharyngeal sample and are able to unveil co-colonization of *S. pneumoniae*, especially in cultures of polymicrobial samples^6,10^.

Direct molecular methods are particularly relevant in case of antibiotic use for respiratory tract infections in the community^11^.

Among culture-independent assays, many researchers opt to use real-time PCR targeting the *lytA* (*lytA*-CDC)^12^, *piaB*^8,13^ and, recently, the SP2020^14^ genes. The latter is a putative transcriptional regulator of the GntR-family, which belongs to the core genome of pneumococcus, and has emerged as an additional candidate gene for accurate pneumococcal identification^14^.

The 7-valent pneumococcal conjugate vaccine (PCV7), which included capsular antigens of *S. pneumoniae* serotypes 4, 6B, 9V, 14, 18C, 19F and 23F was incorporated into the national immunization program (NIP) of Greece in January 2006. Later, the higher-valent PCVs were introduced: the 10-valent (PCV10, with the additional serotypes 1, 5, 7F to PCV7) became available in May 2009 and the 13-valent (PCV13, with further addition of serotypes 3, 6A and 19A to PCV10) in June 2010, and were both incorporated directly into the NIP. Since July 2010, the PCV13 has become the most used PCV^15^, soon increasing to over 97% coverage of PCV vaccinated children. From January 2006 through June 2019, PCVs have been included in the NIP in a 3 + 1 schedule; specifically, immunization at 2, 4, and 6 months of life and a 4th (booster) dose − initially recommended to be administered at 12–18 months, later revised to 12–15 months as of December 2011- have been recommended. Despite the lack of an official vaccination registry in Greece, it is estimated that complete PCV-series coverage is approximately 80% among children < 5 years^16^, our group has previously conducted surveys of pneumococcal carriage among young children in various areas of Greece since 1995^15,17–19^.

The aim of the present cross-sectional study among Greek children was to investigate potential changes of pneumococcal oropharyngeal carriage in the years that follow the 4th dose of PCV13. The choice to sample the oropharynx was made because of the limited molecular data currently available in children on *S. pneumoniae* carriage at this site and the convenience of the approach. We applied molecular methods of carriage detection to assess the patterns of pneumococcal carriage among children of increasing age, including their sixth year of life, who were fully immunized with PCV13 in a 3 + 1 schedule.

## Methods

### Study population

From January 18 to August 29, 2017, children who had completed the 4-dose course of PCV13 immunization, as per the Greek NIP recommendations, were prospectively enrolled by 45 general pediatric practice facilities in a consecutive fashion over 30 municipalities across Greece (Supplementary Fig. S1). A single sample from the posterior pharyngeal wall (oropharynx) was obtained from each subject in a standardized manner (questionnaire, procedure). Children were eligible for inclusion in the study if they: (a) were younger than seven years (≤ 83 months) of age; (b) were not diagnosed with severe chronic lung disease; (c) had received all four doses of the 3 + 1 PCV13 schedule; (d) had received the 4th dose of PCV13 at least 21 days prior to enrollment; and (e) had not received antibiotic treatment within seven days prior to enrollment. Based on the time elapsed between the 4th PCV13 dose and the sampling procedure (time interval post booster of PCV13) children were grouped for analysis into six groups: 26 days (d) to 11 months (m); 12–23 m; 24–35 m; 36–47 m; 48–59 m; and 60–70 m. Presence of respiratory tract infection (RTI) at the time of sampling was also considered. Carriage of *S*. *pneumoniae* and serotyping were evaluated by real-time PCR.

### Questionnaire

The aim and procedures of the study were meticulously explained to the parents/guardians and written informed consent was obtained from at least one guardian of each participating child, attesting (a) acceptance to respond to the administered questionnaire and (b) allowance to obtain an oropharyngeal sample from their child. All methods were performed in accordance with relevant legislative guidelines and regulations. The research protocol was approved by the Ethics Committee of the General University Hospital of Larissa, Greece.

Upon recruitment, the parent(s) responded to an interviewer-administered questionnaire. Information regarding demographic characteristics and immunization with PCV13, including the exact dates of administration, was derived from each child’s health booklet and the interviewing pediatrician’s immunization data base. During the interview, an effort was made to retrieve additional data from the child’s medical files and the responses were recorded on structured report forms.

### Pharyngeal specimen collection

The oropharyngeal sample was obtained from each child at the time of enrollment. A sterile cotton swab was inserted through the mouth and the posterior wall of the oropharynx was sampled. Samples were obtained using an ESwab kit containing a polypropylene screw-capped tube filled with 1 mL of liquid Amies medium (Brescia, Copan, Italy). The sampling was carried out by pressing the tongue downward to the floor of the mouth with a tongue depressor and swabbing the posterior pharyngeal wall without touching the sides of the mouth, the uvula or the tongue. Sample swabs were secured in tubes, stored at 4 °C, and transferred on ice via the Laboratory of the Division of Pediatric Infectious Disease of the University of Thessaly to the Laboratory of the University of Florence and Anna Meyer Children’s Hospital, Florence, Italy on a weekly basis.

### Real-time PCR for the *lytA* gene

Total nucleic acid was automatically extracted from oropharyngeal swab samples, using the MagCore Genomic DNA Tissue Kit with automated Nucleic Acid Extractor HF16 (RBCBioscience, Taiwan) according to manufacturer’s instructions. Extracted DNA from the swab samples were stored at − 20 °C. All DNA samples were tested with real-time PCR for the *lytA* (*lytA*-CDC) gene as previously described^12,20^.

### Singleplex real-time PCR assays for serotypes/serogroups

All *l*yt*A-*positive samples were further molecularly serotyped using singleplex real-time PCR assays for 33 serotypes/groups (1, 2, 3, 4, 5, 6A/B/C/D, 7A/F, 7B/C, 8, 9A/L/N/V, 10A/B, 11A/D/E, 12A/B/F/44/46, 13, 14, 15A/B/C/F, 16F, 17F, 18B/C/F, 19A, 19F, 20, 21, 22A/F, 23A, 23B, 23F, 29, 31, 33F, 35B/D, 35F/37 and 38/25A/F) as previously described^20,21^, also taking into account the following modifications: (a) the primer and probe sets for the serotype/serogroup 7B/C, 31 and 35F/37 have been designed for this study protocol, and (b) the primer and probe sets for the serotype/serogroup 2, 5, 17F, 18B/C/F, 19A, 19F, 22A/F, 35B/D, and 38/25A/F have been redesigned to avoid false-positive signal^10^. All probes are 5’ FAM and 3’ TAMRA labelled (Supplementary Table S1).

Briefly the real-time amplification was performed in 20 µL reaction volumes containing 2× TaqMan Universal Master Mix (Applied Biosystem, Foster City, CA, USA); five µL of DNA extract was used for each reaction. DNA was amplified in an ABI 7500 FAST sequence detection system (Applied Biosystem, Foster City, CA, USA) using, for all the primer pairs, the same cycling parameters as follows: 95 °C for 20 s followed by 45 cycles of a two-stage temperature profile of 95 °C for three s and 60 °C for 30 s. Samples were considered positive for presence of the targeted sequence when the serotype/serogroup specific signal was ≤ 35 Cycle Threshold (CT). If no increase in fluorescent signal was observed after 35 cycles for any of the primer/probe set, the sample was assumed to be negative for the serotype/serogroup specific primers and was reported as not able to be assigned to a specific serotype.

Due to the high genotypic similarities among the capsule loci of certain serotypes, the PCR method was unable to discriminate among certain serotypes within a particular serogroup.

### Procedures applied to reduce risk of false positivity

Two different procedures have been simultaneously applied to reduce the risk of false positivity. First, all samples in which serotype/serogroup CT was lower by > 2 CT than *lytA* CT were eliminated from the analysis of that specific serotype/serogroup as previously discribed^5^. Second, to estimate the background of false positivity, for each serotype/serogroup we tested 100 DNA *lytA*-negative samples using singleplex real-time PCR assays as described above. The rate of false positivity for each serotype/serogroup was then subtracted from positivity rate of each serotype/serogroup (Table 1). Moreover, all serotypes/serogroups showing a false positivity background over 3% were eliminated from analysis, i.e., serotypes/serogroups 4, 5, and 18B/C/F.

**Table 1:** Initial and true (final) *lytA*-positive samples per serotypes/serogroup after subtraction of false positivity (bold).

Serotypes/serogroup	Rate of positivity initially revealed in *lytA-*positive samples (%)	Rate of false positivity revealed in *lytA-*negative samples that had to be subtracted (%)	Rate of true positivity (%)
1	1.5	0.0	1.5
2	0.8	0.0	0.8
3	4.2	0.0	4.2
6A/B/C/D	1.4	0.0	1.4
7A/F	0.0	0.0	0.0
8	0.5	0.0	0.5
9A/L/N/V	2.5	0.0	2.5
10A/B	4.1	0.0	4.1
12A/B/F/44/46	1.5	0.0	1.5
13	0.0	0.0	0.0
14	0.0	0.0	0.0
16F	1.2	0.0	1.2
17F	0.5	0.0	0.5
19A	4.4	0.0	4.4
19F	3.1	0.0	3.1
20	1.7	0.0	1.7
21	5.9	0.0	5.9
22A/F	3.4	0.0	3.4
23A	6.6	0.0	6.6
23B	11.0	0.0	11.0
23F	0.0	0.0	0.0
29	0.0	0.0	0.0
33F	3.1	0.0	3.1
35B/D	4.8	0.0	4.8
35F/37	7.3	0.0	7.3
38/25A/F	4.6	0.0	4.6
**7B/C**	**3.6**	**1.0**	**2.6**
**11A/D/E**	**14.4**	**3.0**	**11.4**
**15A/B/C/F**	**14.8**	**1.0**	**13.8**
**31**	**4.8**	**1.0**	**3.8**

### Real-time PCR for SP2020 gene

*lytA*-positive samples were tested with real-time PCR for SP2020 as previously described^14^. After initial handling and testing, 46 samples did not contain the necessary quantity of material for further analysis. SP2020 CT, similar to *lytA*, was set at ≤ 35.

### Statistical analysis

Pneumococcal isolates were classified as PCV13 serotypes, non-PCV13 serotypes, those belonging to serogroups with both PCV13 and non-PCV13 serotypes and isolates that could not be assigned to a specific serotype/serogroup. To assess the six groups of children who had received the 4th PCV13 dose at different time intervals before the sampling, categorical parameters were compared using 2-sided chi-square test for trend. Results were expressed as median and interquartile range (IQR) or as mean value with 95% confidence intervals (95%CI) as deemed appropriate. For the assessment of two groups, categorical parameters were compared using 2-sided Fisher exact test. In addition to the exploratory analysis, multivariate logistic regression was used to test for the effect of the explanatory independent variables combined. Concordance for CTs between *lytA* and SP2020 genes was assessed by the Intraclass Correlation Coefficient (ICC). All analyses were performed with the IBM SPSS software version 26.0 (IBM Corp., Armonk, NY). Two-sided *P*-values < 0.05 were considered statistically significant.

## Results

### Study demographics

A total of 1256 children/samples were investigated. Forty-four children were excluded as 27 had not received all four doses of PCV13, 15 were ≥ 7 years old and two sample vials were empty. Eligible for further analysis were 1212 samples/children aged 14–83 m. The characteristics of the enrolled attendees appear in the first two columns of Table 2; 34.1% of evaluated children were aged ≥ 60 months. Among children aged 14–23 m 19.9% attended daycare and 46.9% had no siblings, whereas among children aged 24–35 m 61.1% attended daycare and 35.5% had no siblings.

**Table 2:** Characteristics of the study population.

Sample indices	Total samples n (%)	*lytA*-positive samples n (%)	*P*-value	*lytA*-positive samples with typeable isolates n (%)	*P*-value
**Total evaluable children (samples)**	1212	589 (48.6)		483 (39.9)	
**Male gender**	648 (53.5)	321 (54.5)		274 (56.7)	
**Age**					
Median (range), months IQR^a^ (range), months	49 (14–83)	53 (16–83)		53 (17–83)	
	29–66	33–68		34–67	
**Medical condition at time of sampling**					
Healthy Respiratory tract infection	836 (69.0)	387/836 (46.3)	**0.02**	316/836 (37.8)	**0.03**
	376 (31.0)	202/376 (53.7)		167/376 (44.4)	
**Siblings**					
0 ≥ 1 N/A^b^	351 (29.0)	149/351 (42.5)	**0.006**	125/351 (35.6)	0.052
	860 (70.9)	440/860 (51.2)		358/860 (41.6)	
	1 (0.1)	0/1		0/1	
**Daycare or school attendance**					
Yes No N/A	844 (69.6)	461/844 (54.6)	** < 0.001**	388/844 (46.0)	** < 0.001**
	362 (29.9)	126/362 (34.8)		93/362 (25. 7)	
	6 (0.5)	2/6 (33.3)		2/6 (33.3)	
**Antibiotic treatment 8–60 days before sampling**					
Yes No	141 (11.6)	65/141 (46.1)	0.528	54/141 (38.3)	0.688
	1071 (88.4)	524/1071 (48.9)		429/1071 (40.1)	

^a^IQR: interquartile range.

^**b**^Ν/Α: not available.

### Oropharyngeal carriage of *S. pneumoniae*

Of the 1212 children studied, 589 (48.6%) were identified as carriers of *S. pneumoniae* having a *lytA*-positive sample. Serotyping revealed that among carriers 483 (82%) were colonized with typeable isolates. Three hundred and sixty three of 483 (75.2%) children were carriers of a single serotype, while 120 of more than one serotypes (24.8%) (Supplementary Fig. S2). The material of 106 of the total samples (18%) could not be assigned to a specific serotype*.*

**Figure 1:**
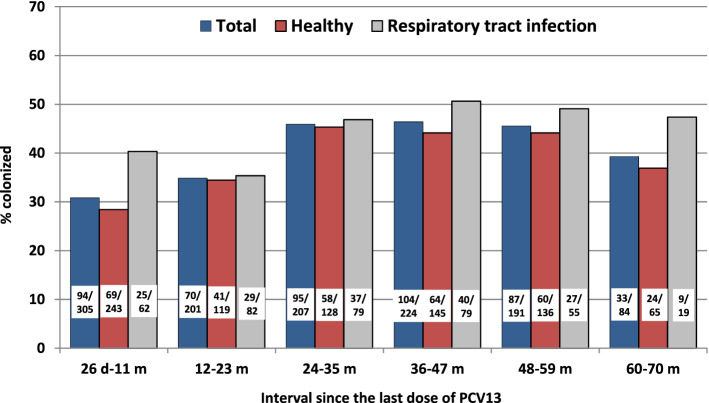
Colonization rate of children with typeable isolates according to increasing interval since the last dose of PCV13 (total, healthy and children with respiratory tract infection).

The last four columns of Table 2 depict the oropharyngeal colonization of all *lytA*-positive samples and typeable *S. pneumoniae* according to the characteristics of our study population. Figure 1 and Supplementary Fig. S3 present the typeable isolates and total *lytA*-positive samples, respectively, according to the post booster time interval; healthy children and those with RTI are also presented. There is a statistically significant increasing trend of *S. pneumoniae* colonization with increasing time interval in both typeable *S. pneumoniae* and total *lytA*-positive samples among healthy children (χ^2^ for trend *P* = 0.002 and *P* < 0.001, respectively). It is also evident from Fig. 1 and Supplementary Fig. S3 that this trend is due to the initial three time interval groups (i.e., 26 d–11 m, 12–23 m and 24–35 m) of healthy children. The same trend is also reflected in the total samples of both typeable and total *lytA*-positive groups (χ^2^ for trend *P* < 0.001; the frequencies appear to plateau in the last four post booster time interval groups of healthy and total sample children. In multivariate analysis of the risk factors presented in Table 2, daycare/school attendance emerges as the main contributing factor of pharyngeal pneumococcal colonization.

When considering the total typeable (n = 483) and total *lytA*-positive samples (n = 589), daycare/school attendees were more frequently colonized with *S. pneumoniae vs.* non-attendees (*P* < 0.001) and this was also the case for non-attendees who had one or more siblings at home (*P* = 0.006 and *P* < 0.001, respectively) as well as children with RTI *vs.* healthy children (*P* = 0.03 and *P* = 0.02, respectively) (Supplementary Fig. S4 and S5).

### Serotypes of interest

**Figure 2:**
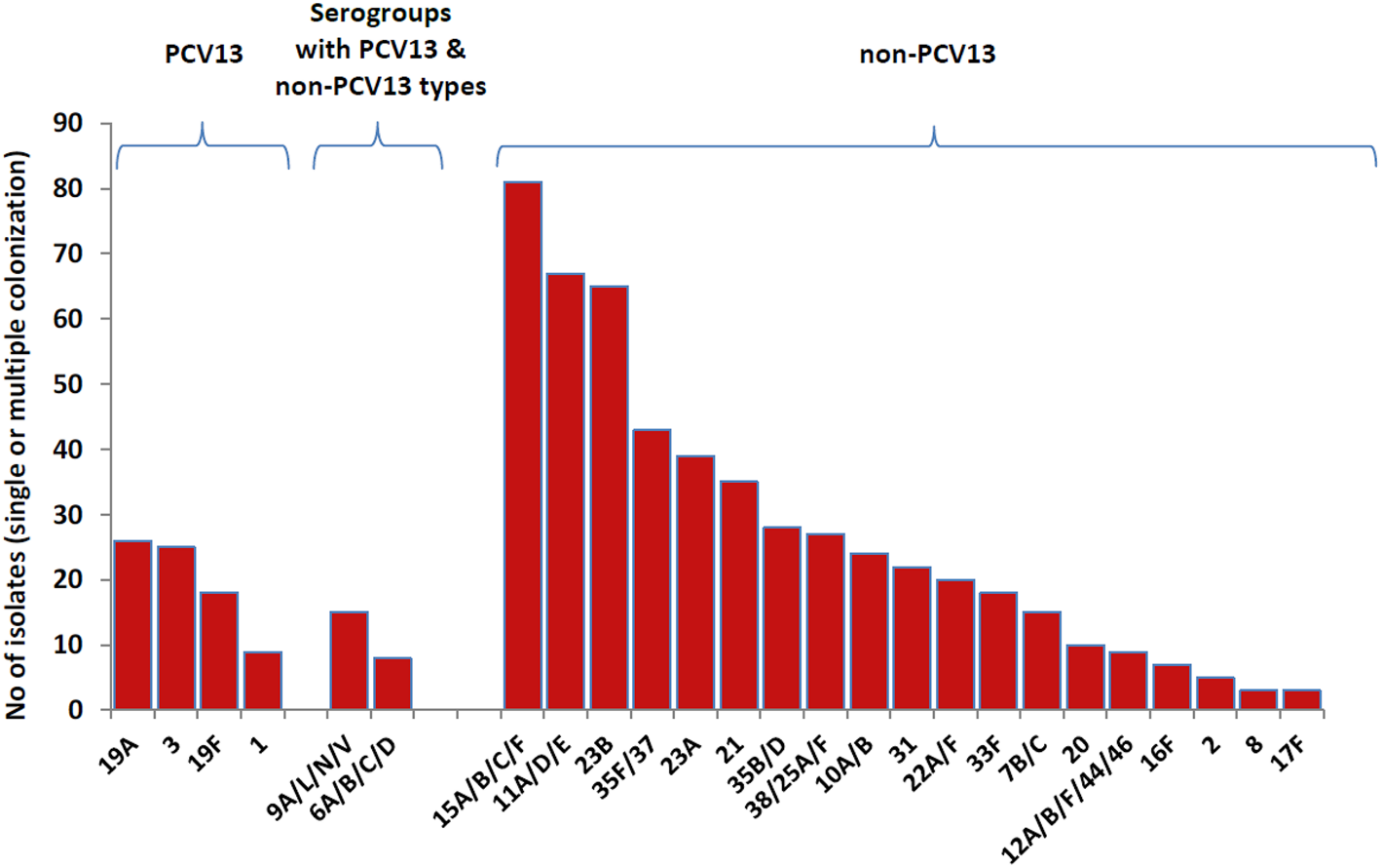
Study serotypes/serogroups revealed according to their inclusion in the PCV13.

Due to co-colonization, a total of 622 *S. pneumoniae* isolates (belonging to 25 serotypes/serogroups) were identified. Of these, 78 (12.5%) belonged to a PCV13 serotype, 23 (3.7%) to serogroups encompassing PCV13 and non-PCV13 serotypes and 521 (83.8%) to a non-PCV13 serotype/serogroup (Fig. 2).

**Figure 3:**
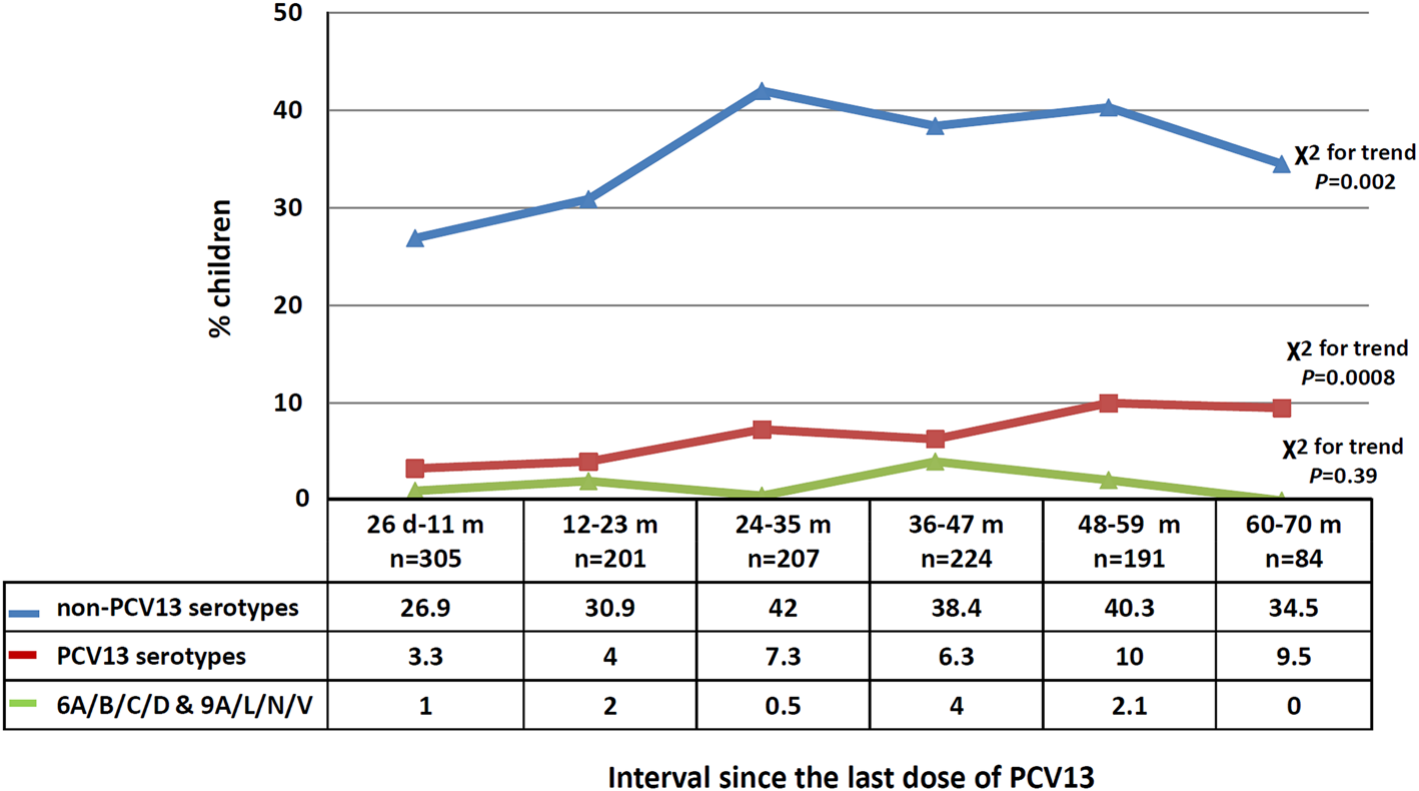
Percentage of colonized children with non-PCV13 serotypes, PCV13 serotypes, and serogroups 6A/B/C/D and 9A/L/N/V according to time elapsed since the 4th (booster) dose. There is significant increase of colonization with non-PCV13 and PCV13 serotypes with increasing time interval.

Figure 3 shows the increase of PCV13 and non-PCV13 serotypes with increasing post booster time interval; there was no such increase in serogroups 6A/B/C/D and 9A/L/N/V which include both PCV13 and non-PCV13 serotypes.

**Figure 4:**
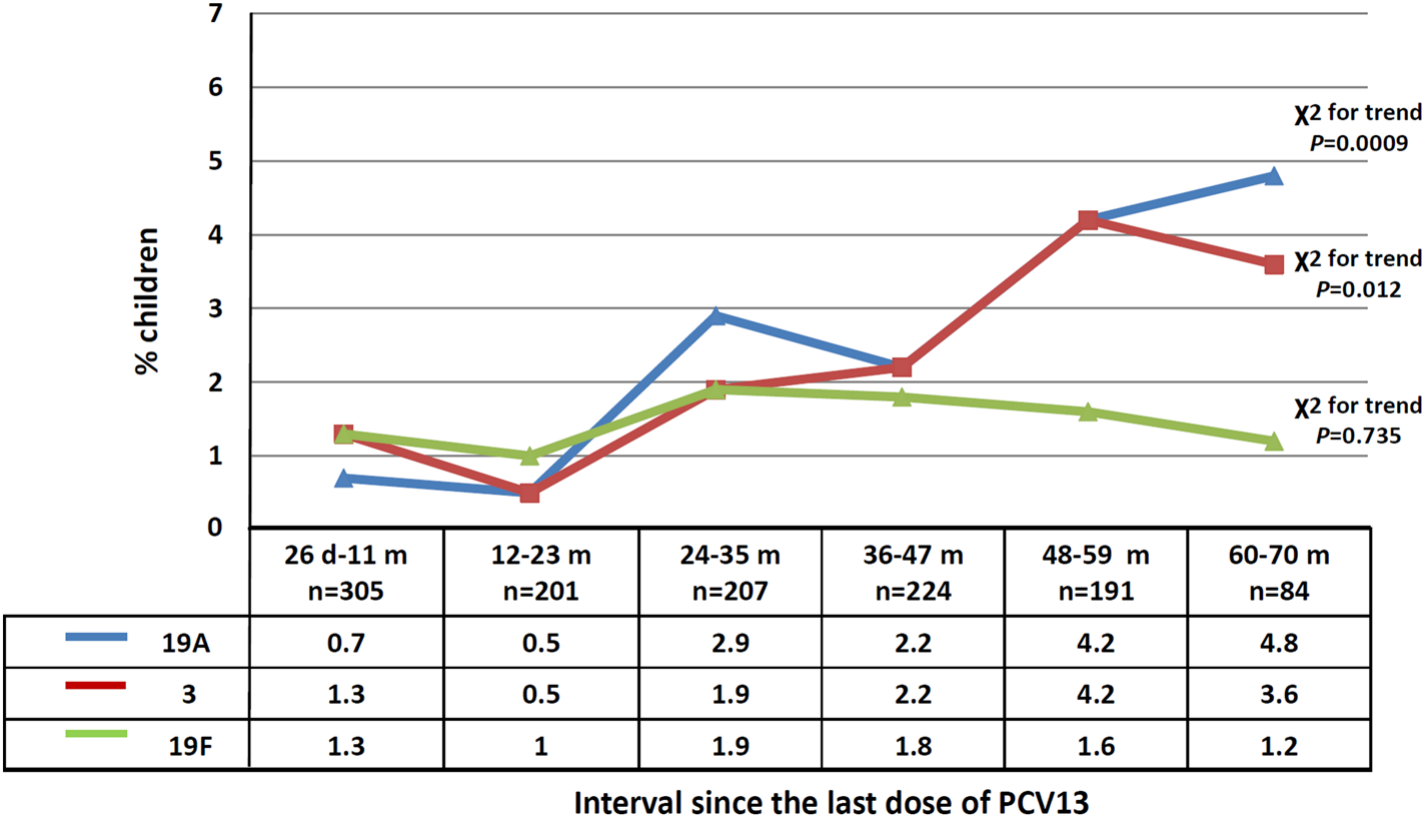
Percentage of colonized children with serotypes 19A, 3 and 19F according to time elapsed since the 4th (booster) dose. There is a significant increase of colonization of serotypes 19A and 3 with increasing time interval.

Pneumococci belonging to serotype 19A were recovered from 26 carriers (living in 12 municipalities), serotype 3 from 25 carriers (15 municipalities) and serotype 19F from 18 carriers (11 municipalities). Serotypes 3 and 19A were the only PCV13 serotypes in which the percentage of colonization increased significantly as post booster time interval increased (Fig. 4). There was no increase in 19F carriage (Fig. 4), and there was no child carrying serotypes 23F, 14 and 7F.

### SP2020 gene analysis

**Figure 5:**
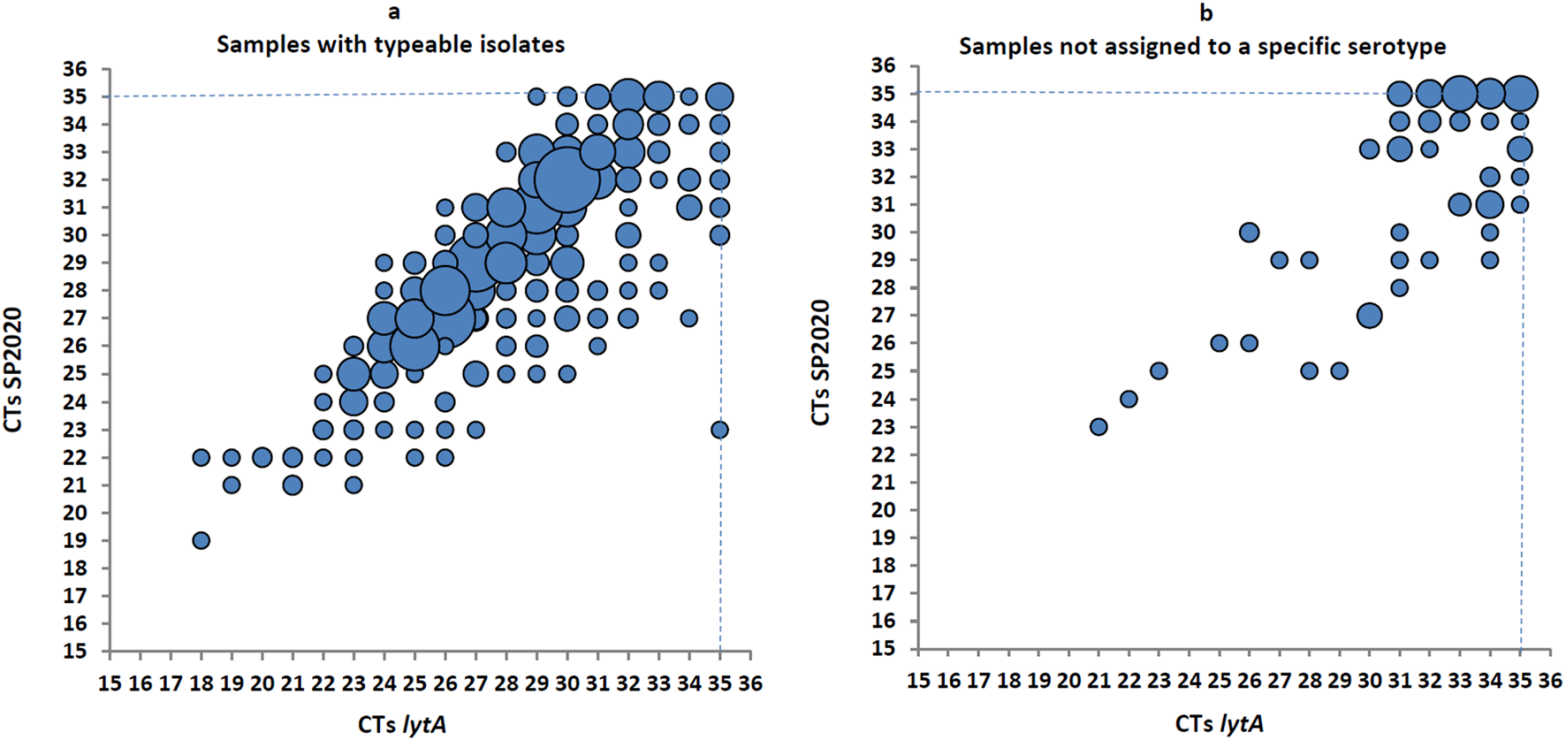
Samples tested positive for both genes: typeable (n = 424) (**a**) and samples not assigned to a specific serotype (n = 84) (**b**). Each blue circle indicates the CT values of samples tested positive for both *lytA* and SP2020 genes. The size of the blue circles (‘bubbles’) is proportionate to the number of samples which presented with the specific CT combination.

Of the 483 typeable samples, 437 were available for testing for the SP2020 gene*.* Of these, 424 (97.0%) were positive for both *lytA* and SP2020 gene; the 13 typeable *lytA*-positive, but SP2020-negative isolates belonged to serotypes/serogroups 2 (n = 1), 3 (n = 1), 11A/D/E (n = 1), 12A/B/F/44/46 (n = 1), 15A/B/C/F (n = 2), 16F (n = 1), 19F (n = 1), 20 (n = 1), 23B (n = 2), 31 (n = 1) and 35B/D (n = 1). Of the 106 *lytA*-positive samples that could not be assigned to a specific serotype, 84 (79.2%) were positive for the SP2020 gene. The SP2020 positivity difference between typeable and *lytA*-positive isolates that could not be assigned to a specific serotype is significant (*P* < 0.001). The CTs of the respective real-time PCR are presented in Fig. 5.

The concordance between CTs of the two genes was: mean 0.86; 95%CI 0.76 to 0.91 (*P* < 0.001) for typeable isolates (Fig. 5a), and 0.83; 95%CI 0.73 to 0.88 (*P* < 0.001) for isolates which could not be assigned to a specific serotype/group (Fig. 5b). When the total number which included both *lytA*-positive groups was analyzed the ICC rendered: mean 0.87; 95%CI 0.80 to 0.81.

**Figure 6:**
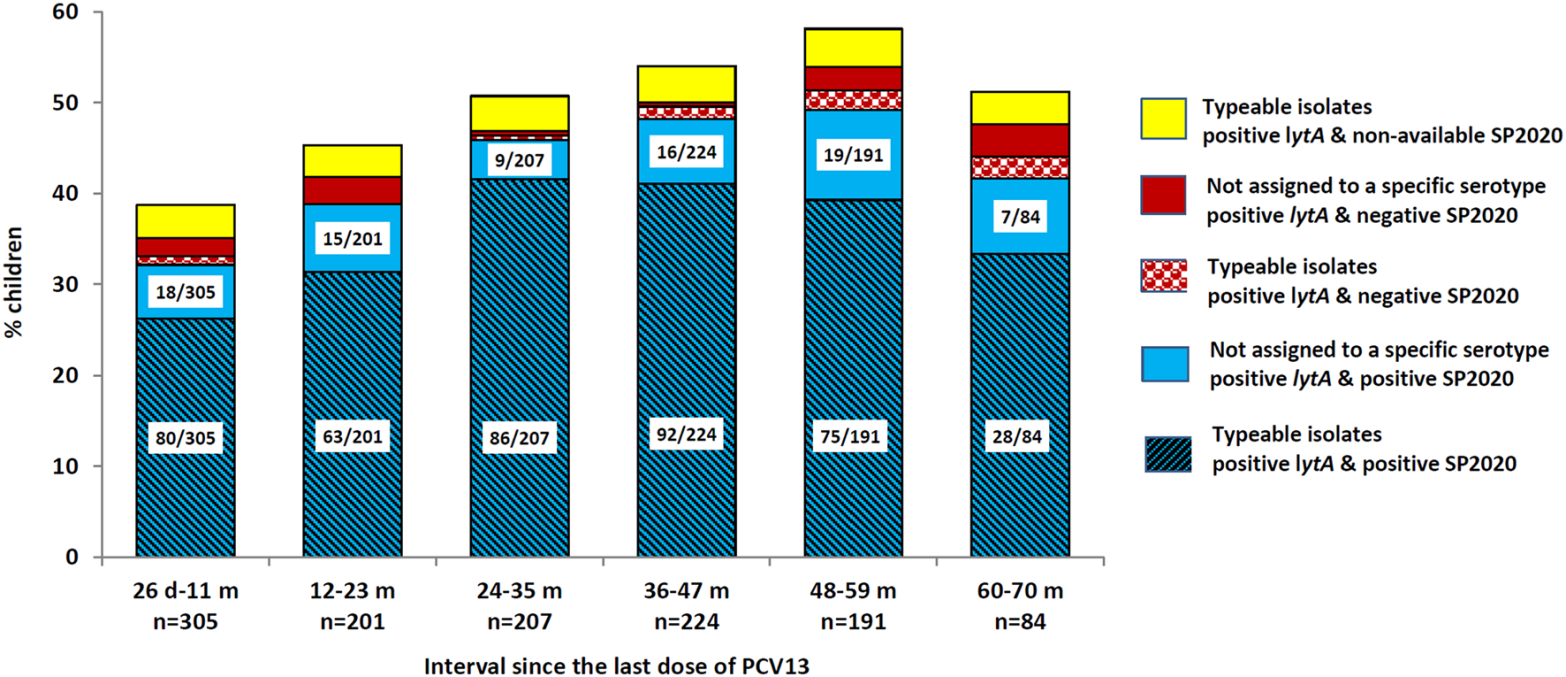
*lytA* and SP2020 gene positivity status of samples, according to time elapsed since the 4th (booster) dose (six interval durations spanning 26 days to 70 months).

The sample analysis as per both *lytA* and SP2020 genes according to post booster time interval is presented in Fig. 6. There is a statistically significant increasing trend of *S. pneumoniae* colonization with increasing post booster time interval in both typeable *S. pneumoniae* and total *lytA*- and SP2020-positive samples (χ^2^ for trend *P* = 0.001 and *P* < 0.001, respectively); no such trend was observed among isolates which could not be assigned to a specific serotype/serogroup (*P* = 0.146).

The CTs of *lytA* and SP2020 real-time PCRs of the two most common PCV13 serotypes, i.e., 19A and 3, and the two most common non-PCV13 serogroups, i.e., 15A/B/C/F and 11A/D/E are presented in Supplementary Fig. S6.

Co-colonization with two or more serotypes of *lytA*-positive samples over increasing post booster PCV13 time intervals showed an increasing trend (χ^2^ for trend *P* < 0.001); this was also the case among *lytA*- and SP2020-positve samples (χ^2^ for trend *P* = 0.002) (Supplementary Table S2).

## Discussion

In the present cross-sectional study, with the use of molecular methods, we aimed to gain insight into *S. pneumoniae* colonization in a large sample of Greek children, who were fully immunized with PCV13 in a 3 + 1 immunization schedule over an age range of 14 through 83 months. The sample collection spanned over three seasons: half of winter, spring, and summer of 2017. An experienced group of investigators, who have previously conducted similar surveillance studies, was able to apply real-time PCR in order to compare *S. pneumoniae* colonization in various age-groups, at increasing time intervals after administration of the 4th (booster) PCV13 dose^22,23^. We found an overall *lytA*-positive carriage rate of 48.6% in our total oropharyngeal sample in a childhood population of small sized families, with relatively late entrance to daycare/school, primarily followed in pediatric private practice. Eighty-two percent were typeable isolates, of which 83.8% consisted of exclusively non-PCV13 serotypes. Co-colonization was observed in 24.8% of children. In univariate analysis there was an increasing time trend in the frequency of colonization and co-colonization for increasing time interval from completion of PVC13 immunization (four doses) among healthy children but not in those with RTI. Children attending daycare/school, those not attending but with one or more siblings at home, and those with RTI at the time of sampling were at increased risk of *S. pneumoniae* colonization. Importantly, an increasing trend of PCV13 serotypes 19A and 3 with increasing time interval from completion of full vaccination groups emerged.

If *lytA*-positive samples that could not be assigned to a specific serotype/serogroup were not included in the analysis, only slight differences in the study results were observed. These excluded *lytA*-positive samples most likely consisted of a mixed group of true non-typeable (non-encapsulated) pneumococcal isolates, serotypes not identified by our molecular assays, and non-pneumococcal streptococci^24^. The exact contribution of each of these three groups remains unknown.

It is widely appreciated that the use of conventional culture-based techniques and serotyping of pneumococci by antisera lack sensitivity in detecting *S. pneumoniae* isolates and render lower bacterial density in the upper airway^6,10^. We applied real-time PCR using the *lytA*-CDC gene which is the recommended method for detecting *S. pneumoniae*^12^. To obtain true positive results, the cut-off for real-time PCR when targeting the *lytA* gene was set at ≤ 35 CTs. Two further procedures were applied simultaneously to reduce the risk of false positivity. First, all samples in which serotype/serogroup CT was lower by > 2 CT than *lytA* CT were eliminated from the analysis of that specific serotype/serogroup as previously discribed^5^. Second, to estimate the background of false-positivity, for each serotype/serogroup we tested 100 DNA *lytA*-negative samples using singleplex real-time PCR assays as described above. The rate of false positivity for each serotype/serogroup was then subtracted from the positivity rate of each serotype/serogroup (Table 1). Moreover, all serotypes/serogroups showing a false positivity background over 3% were eliminated from analysis. We trust that the combination of these methodological precautions offer reasonable assurance of the true presence of *S. pneumoniae* in our samples and the subsequent analysis of our results. Furthermore, based on DNA sequence differences between pneumococcal *lytA* and its homologues, real-time PCR assays for specific identification of pneumococcus have been developed^12^.

Tavares et al. have proposed that, in adults, combined use of both *lytA*-CDC and the SP2020 gene is a powerful strategy for the identification of pneumococcus, in both pure cultures and polymicrobial samples^14^. In addition to *lytA* we applied the SP2020 gene on the available *lytA*-positive samples (92.2%). Ninety-seven percent of our typeable samples were positive for both genes, whereas this percentage dropped to 79.2% when the SP2020 gene was applied to those that could not be assigned to a specific serotype/serogroup.

Current real-time PCR assays are not optimized to detect all possible pneumococcal serotypes. Therefore, a proportion of samples that cannot be assigned to a specific serotype/serogroup are not detected, leading to underestimation of the frequency of serotype colonization. This is also the case with our study. In addition, our analysis was hampered by the inability to assign specific serotypes within serogroup 6, which includes serotypes 6A, 6B, 6C and 6D, and serogroup 9, which includes 9A, 9L, 9N and 9V. Both PCV13 and non-PCV13 serotypes are included in these two groups (3.7%), which are depicted as a separate category in Fig. 2. It has been shown that in case of serogroup 6, serotypes 6A and 6B which are included in PCV13, are expected to generate protective antibodies towards 6C and 6D^25–27^; however, this does not appear to be the case with serotype 9V (also included in PCV13), which does not offer cross-protection towards 9L and 9N^28–31^.

Although cross-sectional studies cannot resolve the direction of causality between related events, the choice of our population sample, in conjunction with the use of a molecular approach to investigate pneumococcal carriage, has rendered epidemiologically and clinically useful information. Our study population consisted of a large number (n = 1212) of fully vaccinated children spanning an age range of approximately six years. Potential risk factors such as occurrence of RTI during sampling, history of daycare/school attendance and sibship status at home were considered.

A single oropharyngeal sample was obtained from each child. Both nasopharynx and oropharynx constitute polymicrobial sites, although the oropharynx is characterized by greater bacterial diversity^32^. Currently, sampling of the upper respiratory tract ideally should include both the nasopharynx and the oropharynx; when only one sample is obtained, nasopharyngeal sampling has been suggested as the preferred choice^9^.

In our population, the overall pneumococcal carriage rate (48.6%) was comparable to that reported by other studies evaluating nasopharyngeal samples from vaccinated children of similar age^33–35^.

We observed an increasing trend for both PCV13 and non-PVC13 serotype carriage with increasing time-elapsed post-immunization groups. Non-PCV13 serotypes predominated in all groups and non-PCV13 serotype colonization reached 83.8% in the total isolates. This is consistent with the findings of other studies in similar settings^33–35^. The overall serotype distribution among carriers in our study was similar regardless of the time elapsed since the last PCV13 dose. These findings most likely reflect the full benefit of both vaccine protection and herd immunity, and offer epidemiologically relevant information regarding pneumococcal carriage among children up to 70 months post completion of PCV13.

It should be noted that the overall distribution profile when applying the SP2020 gene on *lytA*-positive typeable samples closely resembles that of the *lytA*-positive alone time trend (Supplementary Fig. S7). The similarity of findings by the two genes reinforces the validity of our results.

The 10 most prevalent non-PCV13 serotypes/serogroups in this study, in order of decreasing frequency, were 15A/B/C/F, 11A/D/E, 23B, 35F/37, 23A, 21, 35B/D, 38/25/A/F, 10A/B and 31. Several of these serotypes/serogroups were also among the most common non-PCV13 ones observed in recent studies evaluating nasopharyngeal carriage of *S. pneumoniae*: nine in the Kandasamy et al*.* (aged 13–48 months 48.7% carriage rate^35^), eight in the Løvlie et al*.* (aged 8 to 80 months 48.1% carriage rate^34^), and seven in the Southern et al*.* (< 5 years 51.9% carriage rate^33^) study, differing only in the ranking of frequency. Notably, the majority of the 10 most prevalent non-PCV13 serotypes/serogroups of this study were also among the 10 most common non-PCV13 serotypes in young carriers in Greece in the late PCV7 and the early PCV13 usage period^15^.

Interestingly, there appears to be a decrease in non-PCV13 carriage when children reach the age of six years and begin attending primary school. Since we did not obtain data beyond this age, this ‘decrease’ could be assigned to a spurious fluctuation of pneumococcal carriage over time-elapsed post-immunization curves; however, it may also, reflect the turning point of a commencing decrease in pneumococcal carriage at school age. Wyllie et al. have shown that use of two-gene quantitative PCR on enriched samples of saliva -also a highly polymicrobial upper respiratory tract source- rendered a 25% decrease of *S. pneumoniae* carriage rate among schoolchildren of an older (8–10 years) *vs.* younger (5–8 years) age^5^. Monitoring of carriage by consistent molecular methods over a longer period, extending into school age and adolescence, will be required to definitively answer this question.

We paid particular attention to serotypes 19A and 3; these were the only two PCV13 serotypes the colonization rate of which increased as time-elapsed post immunization progressed, and both serotypes were identified in several municipalities throughout the country; attendance of daycare/school is probably the most likely explanation. In a recent study from the UK serotypes 19A and 3 continued to circulate among PCV13 immunized children aged 24 to 48 months^35^. In our study, a substantial reduction of carriage rate of other PCV13 serotypes was noted and serotypes 7F, 14 and 23F were absent.

Apart from its cross-sectional design, our study has other limitations. First, to assure recruitment rates and homogeneity of the sampling procedure we opted to sample the oropharynx which is a much easier procedure than nasopharyngeal sampling. Although the oropharyngeal samples are regarded as more polymicrobial as compared to the nasopharyngeal ones^32^, the consistency of our results when applying a second target gene is encouraging in postulating that the oropharynx offers a reliable alternative to estimate pneumococcal carriage in epidemiological studies. Second, the use of the SP2020 target solely on *lytA*-positive samples precludes any comparison of sensitivity and specificity of the two genes; such comparison was not an aim of our study. Third, cost and laboratory time constraints did not allow for the application of Llull’s et al. method of separating pneumococci from mitis group streptococci^36^ and the Multilocus Sequence Typing (MLST) which characterizes *S. pneumoniae* isolates by their unique allelic profiles in our *lytA*- and/or SP2020-positive samples which could not be assigned to a specific serotype; thus, the distribution of 106 unassigned samples into true or false positive *S. pneumoniae* cannot be answered. Fourth, the description of seasonality was not a target of this study and no pharyngeal sampling was performed in autumn as well as in approximately half of the winter seasons; therefore, the sampling season was not accounted for in the multivariate analysis of our results.

In conclusion, we conducted a large cross-sectional study of *S. pneumoniae* oropharyngeal carriage, over 30 municipalities scattered throughout Greece, in which sampling was performed during a time interval of 26 days to 70 months after administration of the 4th (booster) PCV13 dose. By employing real-time PCR targeting the *lytA* gene and using carefully selected control procedures to avoid false positive results, we showed that an overall carriage rate of 48.6%; 83.8% of isolates consisted exclusively of non-PCV13 serotypes and 3.7% of serogroups 6 and 9, which are a mix of PCV13 and non-PCV13 serotypes. There was an increasing trend for carriage with increasing time interval since completion of the vaccination schedule. Notably, serotypes 19A and 3 were the only two PCV13 serotypes the colonization rate of which increased over time. The application of SP2020 as a second target gene on our *lytA*-positive samples rendered an overall colonization profile over time which closely resembled that of the *lytA* gene when used alone. With the provision of the methodological approach and age group of our study and in conjunction with standardized PCR analysis, it offers further support to the value of oropharyngeal sampling in the epidemiological assessment of pneumococcal colonization of the upper airway.

## Supplementary Information

Supplementary Information.

## Data Availability

Basic data analyzed during this study are included in this published article (and its Supplementary Information files) while part of datasets generated during and/or analyzed during the current study are available from the corresponding author upon reasonable request.
