# Supplementary material for: Molecular surveillance of pneumococcal carriage following completion of immunization with the 13-valent pneumococcal conjugate vaccine administered in a 3 + 1 schedule
Source: Sci Rep. 2021 Dec 30;11:24534. doi: 10.1038/s41598-021-03720-y (PMC8718523; doi:10.1038/s41598-021-03720-y)
Supplement: Supplementary file 1 — Supplementary Information. [file 41598_2021_3720_MOESM1_ESM.pdf]

SUPPLEMENTARY INFORMATION

Molecular Surveillance of Pneumococcal Carriage Following Completion of Immunization with the 13-Valent Pneumococcal Conjugate Vaccine Administered in a 3+1 Schedule

George A. Syrogiannopoulos[1], M.D., Ioanna N. Grivea[1], M.D., Maria Moriondo[2], PhD, Francesco Nieddu[2], PhD, Aspasia N. Michoula[1], M.D., Maria Rita Calabrese[2], BSc, PhD, Michael Anthracopoulos[3], M.D., Chiara Azzari[2], M.D.

[1]Department of Pediatrics, University of Thessaly,

School of Health Sciences, Faculty of Medicine, Larissa, Greece

[2]University of Florence and Anna Meyer Children's Hospital, Florence, Italy

[3]Department of Pediatrics, University of Patras, Patras, Greece

Content

Supplementary Figure S1. Map of Greek municipalities from which children were enrolled.

Supplementary Figure S2. *S. pneumoniae* colonization according to the number of serotypes revealed.

Supplementary Figure S3. Colonization rate of total *lytA*-positive samples according to increasing interval since the last (booster) dose of PCV13 (total, healthy and children with respiratory tract infection).

Supplementary Figure S4. Frequency of *S. pneumoniae* colonization in typeable samples according to daycare/school attendance, non-attending with ≥1 siblings at home, and presence of respiratory tract infection.

Supplementary Figure S5. Frequency of *S. pneumoniae* colonization in total *lytA*-positive samples according to daycare/school attendance, non-attending but with ≥1 siblings at home, and presence of respiratory tract infection.

Supplementary Figure S6. *lytA* and SP2020 CTs in samples where the two most common PCV13 serotypes, 19A and 3, and the two most common non-PCV13 serogroups, 15A/B/C/F and 11A/D/E, were found.

Supplementary Figure S7. Percentage of children colonized with non-PCV13 serotypes, PCV13 serotypes, and serogroups 6A/B/C/D and 9A/L/N/V when applying the SP2020 gene on *lytA*-positive samples according to time elapsed since the 4th (booster) dose.

Supplementary Table S1. Primers and probes used for serotype identification.

Supplementary Table S2. Frequency of co-colonization, according to testing by one or two genes.

Supplementary Figure S1. Map of Greece depicting the municipalities of study enrollment. Thirty municipalities –45 general pediatric practices– where children <7 years of age who had completed a 3+1 schedule of pneumococcal vaccination were enrolled. A total of 1256 samples were initially obtained of which 1212 fulfilled the study criteria for analysis (sketch of map from slideshare.net at URL https://image.slidesharecdn.com/4-110727160904-phpapp01/95/4-1-1024.jpg?cb=1573656249, modified for study purposes).

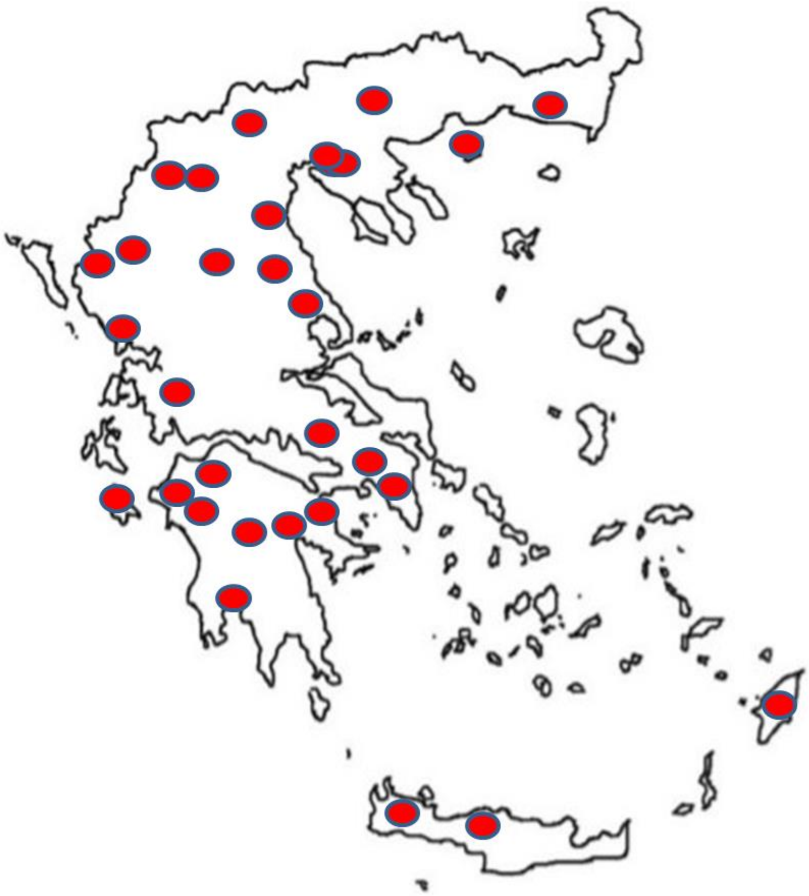

Supplementary Figure S2. *S. pneumoniae* serotype colonization according to the number of serotypes revealed.

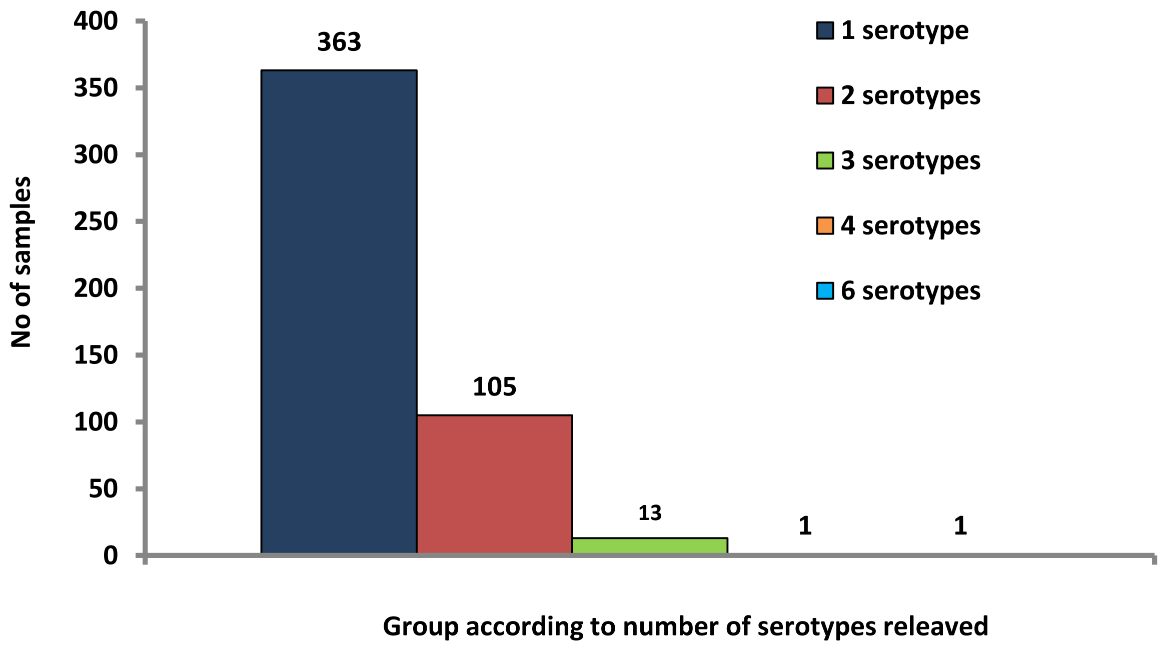

Supplementary Figure S3. Colonization rate of total *lytA*-positive samples according to increasing interval since the last (booster) dose of PCV13 (total, healthy and children with respiratory tract infection).

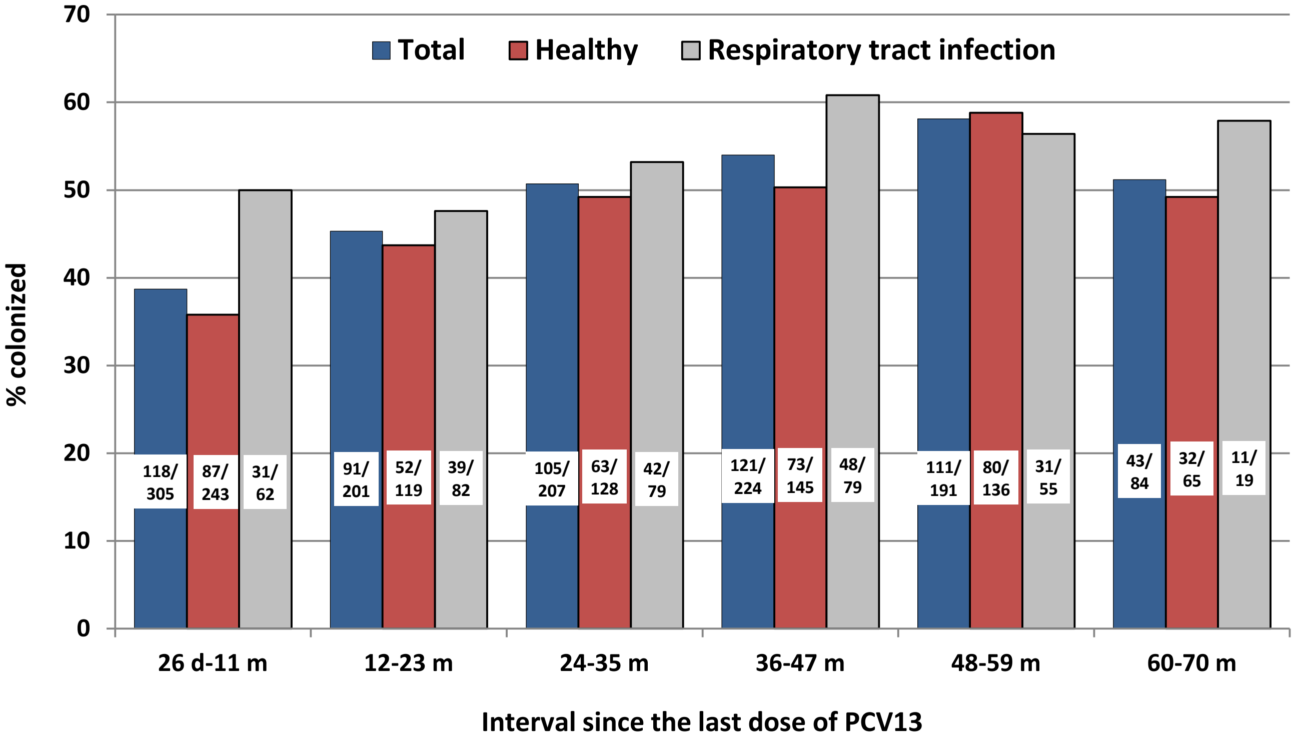

Supplementary Figure S4. Frequency of *S. pneumoniae* colonization in typeable samples according to daycare/school attendance, non-attending but with ≥1 siblings at home, and presence of respiratory tract infection.

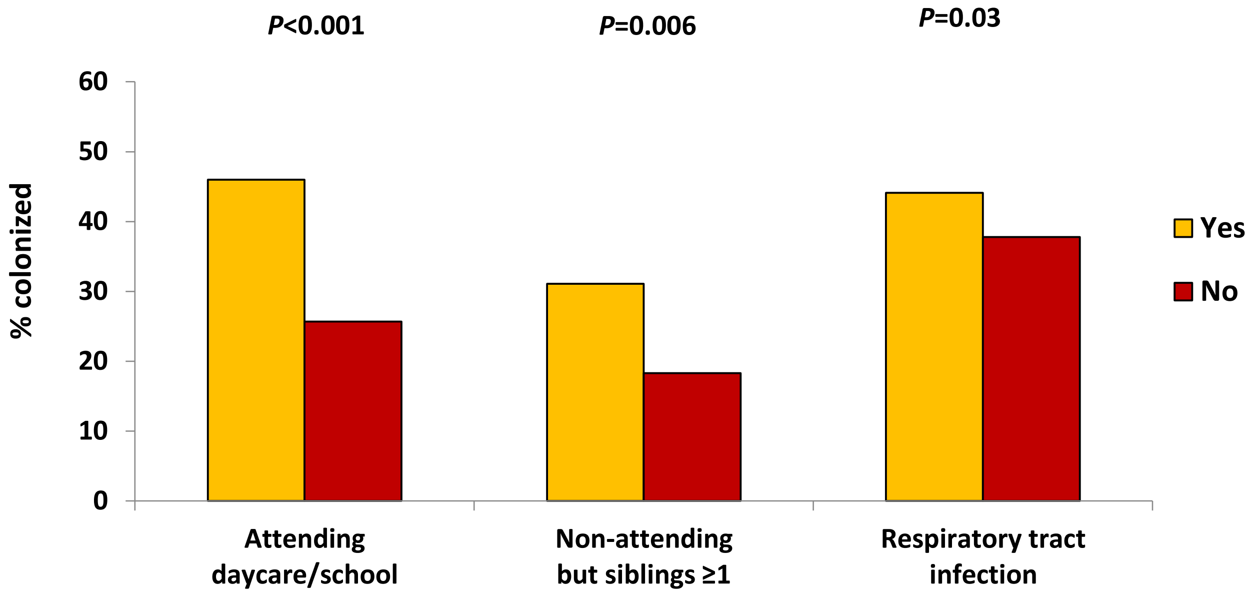

Supplementary Figure S5. Frequency of *S. pneumoniae* colonization in total *lytA*-positive samples according to daycare/school attendance, non-attending but with ≥1 siblings at home, and presence of respiratory tract infection.

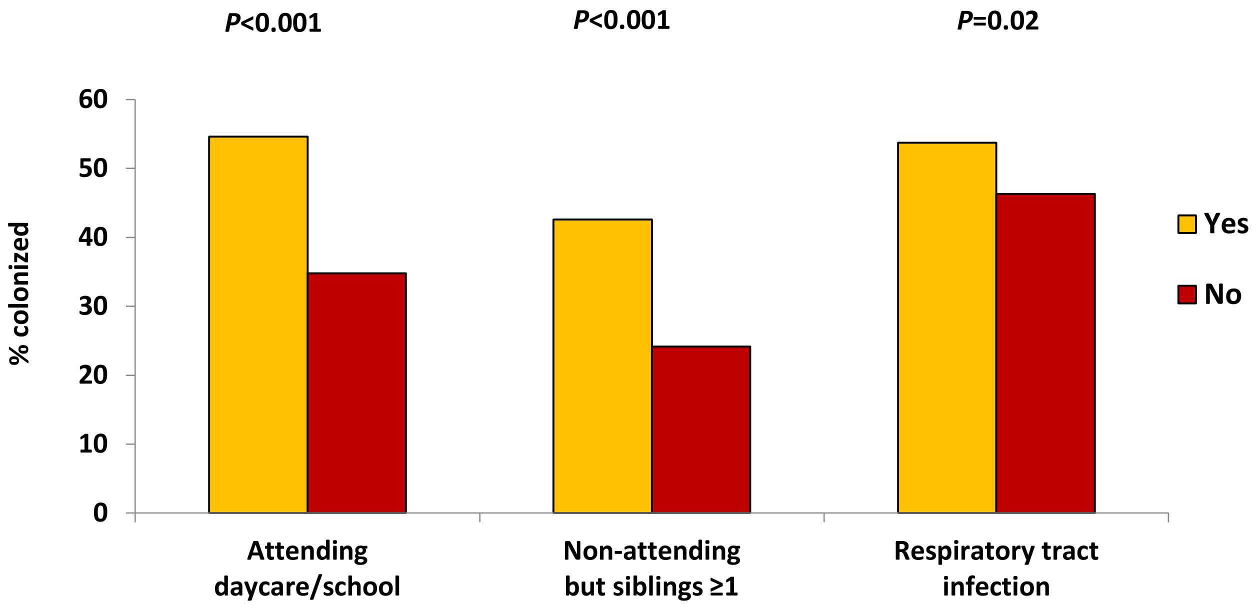

Supplementary Figure S6. *lytA* and SP2020 CTs in samples where the two most common PCV13 serotypes [19A (S6**a**) and 3 (S6**b**)], and the two most common non-PCV13 serogroups [15A/B/C/F (S6**c**) and 11A/D/E (S6**d**)] were found.

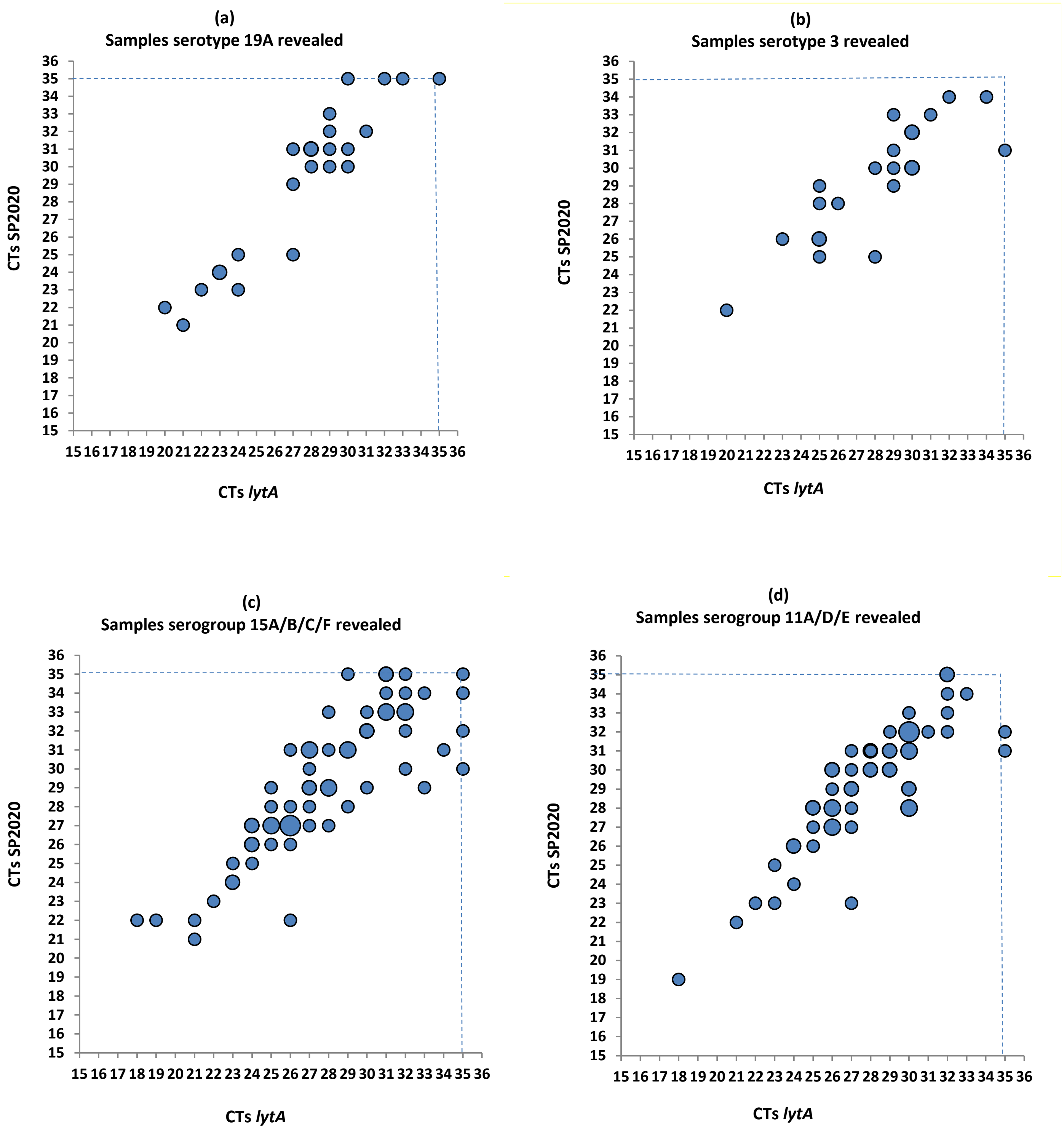

Supplementary Figure S7. Percentage of children colonized with non-PCV13 serotypes, PCV13 serotypes, and serogroups 6A/B/C/D and 9A/L/N/V when applying the SP2020 gene on *lytA*-positive samples according to time elapsed since the 4th (booster) dose.

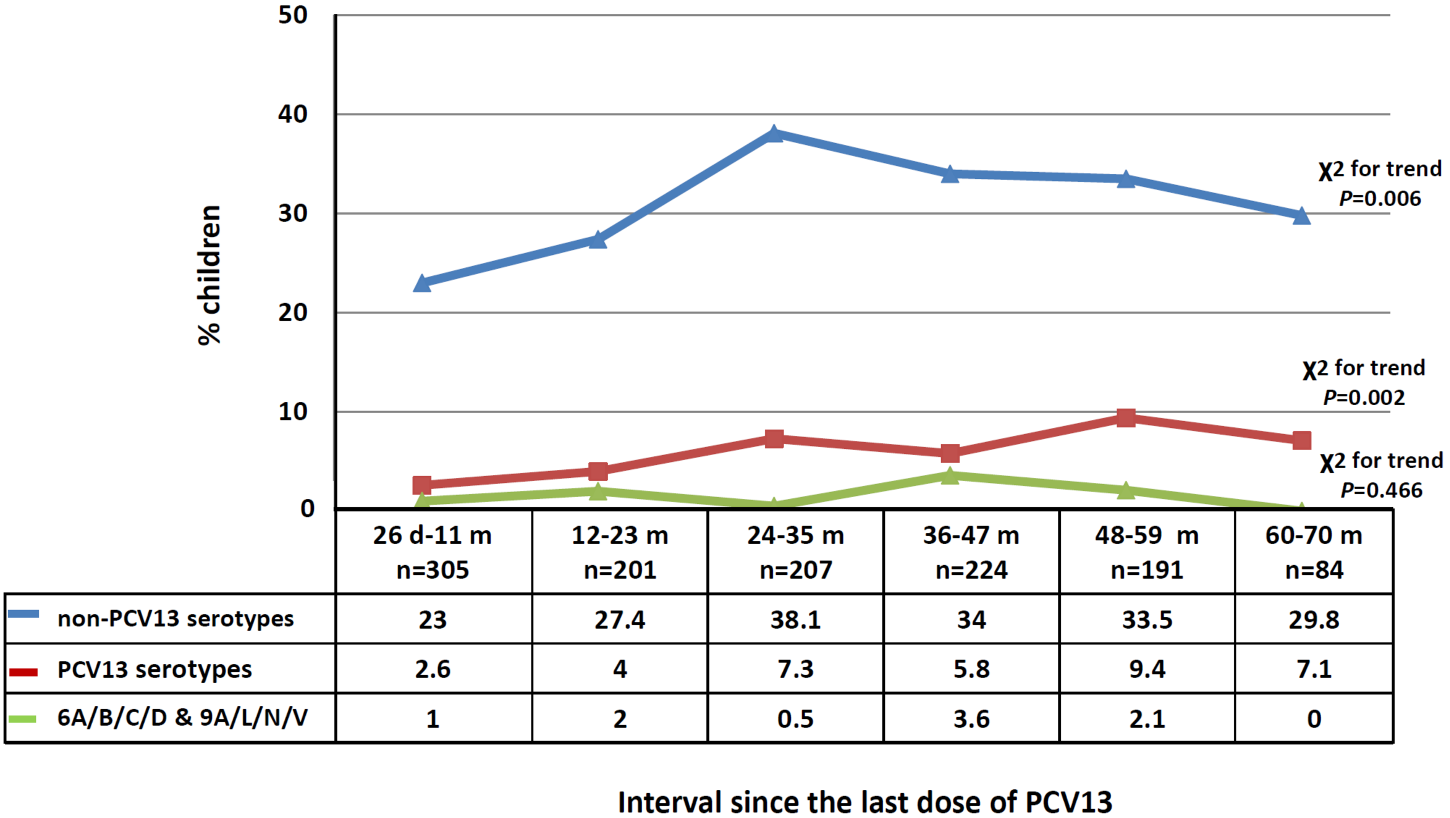

	26 d-11 m n=305	12-23 m n=201	24-35 m n=207	36-47 m n=224	48-59 m n=191	60-70 m n=84
non-PCV13 serotypes	23	27.4	38.1	34	33.5	29.8
PCV13 serotypes	2.6	4	7.3	5.8	9.4	7.1
6A/B/C/D & 9A/L/N/V	1	2	0.5	3.6	2.1	0

Interval since the last dose of PCV13

Supplementary Table S1. Primers and probes used for serotype identification**.** Novel (including those redesigned) primers/nucleotides are presented as grey cells.

Target Name	Forward primer	Reverse primer	probe
lytA	TCTTACGCAATCTAGCAGATGAAGC	GTTGTTTGGTTGGTTATTCGTGC	TTTGCCGAAAACGCTTGATACAGGG
Streptococcus pneumoniae serotype 1	CGT GCG GTA ATT GAA GCT ATG A	TGT GGC CCC AGC AAC TCT	CGT GCT TGC CCT TGT ATA GGG T
Streptococcus pneumoniae serotype 2	GAAAATTGTTTGCCTTAAAGAACCA	TGTGTCGCCCCCACAAC	TTAAATATCGTCAACACGGTGGGAA
Streptococcus pneumoniae serotype 3	GGT CAG CAG AAA GTA TGC ATT GG	TCG TTT ATC CAG GGT CTG ATG A	TAT TGG ATG TGG TTT ATC GTG AAG A
Streptococcus pneumoniae serotype 4	TGG GAT GAC ATT TCT ACG CAC TA	CCG TCG CTG ATG CTT TAT CA	TCC TAT TGG ATG GTT AGT TGG TGA
Streptococcus pneumoniae serotype 5	CAG ATT ATG TGC CTA GTT TAT CGC TCT AT	AAG TGA ATG AGG AAT CAG ATA TTG GTA A	CCT TGC TGC CTG GAG TGG AAC GAT
Streptococcus pneumoniae serogroup 6A/B/C/D	AAG TTT GCA CTA GAG TAT GGG AAG GT	ACA TTA TGT CCR TGT CTT CGA TAC AAG	TGT TCT GCC CTG AGC AAC TGG
Streptococcus pneumoniae serogroup 7B/C	TTCAACAACGGAAGGTTTTGG	TTCTGGACTAGCTCCATTTTTACTTG	TTGTTGAATATTTAGCGAACGGTCTTTGTGTTG
Streptococcus pneumoniae serogroup 7A/F	GAT GGC ATG TGG CAA ACC A	TTT GCC CTC CTT AAT CAT TTC AC	TTG GCT ATC GGC ATG GTG GT
Streptococcus pneumoniae serotype 8	CCA CTC ATC AGT TTC CCA TAT GTT T	TCA ATA ATT GAA GAA GCG AAC GTT	TGA TGG CAG ATG GGT TGG GAC GA
Streptococcus pneumoniae serogroup 9A/L/N/V	TGG AAT GGG CAA AGG GTA GTA	TCG GTT CCC CAA GAT TTT CTC	TTA ATC ATG CTA ACG GCT CAT CGA
Streptococcus pneumoniae serogroup 10A/B	CCT CTC CTA TCA ACT ATT ACT CAT TAT ACT ACC T	AAT AAC CAT AAG TCC CTA GAT CAT TCA AA	TCA TTA CAA CTC CCT ATG TGA CAC GGG TCT TTT
Streptococcus pneumoniae serogroup 11A/D/E	AGCTATCCTTTAGGCATTCCGTTA	TCCCGTTGGCTTAGATATGTGTT	TTGAAACACTAGATGAACTGGCAAACCT
Streptococcus pneumoniae serogroup 12A/B/F/44/46	GAT TAT TCG CTT GCC TCT TCA TG	ATA GCC GAA ATA AGC TTT CCA GAA	ATT TGT AAG CGG ACG TGC GAT T
Streptococcus pneumoniae serotype 13	ATATCCTTATGACCCTGGTAACTTCTTT	ACCGCTTTTTGTTCCCCTTT	TGTTACCTCGTGTTGCGCATCTGCTATC
Streptococcus pneumoniae serotype 14	CGA CTG AAA TGT CAC TAG GAG AAG AT	AAT ACA GTC CAT CAA TTA CTG CAA TAC TC	TGT CAT TCG TTT GCC AAT ACT TGA TGG TCT C
Streptococcus pneumoniae serogroup 15A/B/C/F	TTG AAT CAG GTA GAT TGA TTT CTG CTA	CTC TAG GAA TCA AAT ACT GAG TCC TAA TGA	CTC CGG CTT TTG TCT TCT CTG T
Streptococcus pneumoniae serotype16F	CAGGCGAAAAGCGAGCAT	TGGGTTCCCCTCATCTACGTT	TGCTTTGGTAGCTTGTATGAGTGC
Streptococcus pneumoniae serotype17F	CATATGGAAAAGCACGAGAGAAAA	TCGAGCCCCAACTTTAAGTAGAA	TCCAGTTTACCGATCTGTGAAATCT
Streptococcus pneumoniae serotype 18B/C/F	AGGTACAGCAAATCGAGCTAATACTTT	TTGGGCAACATTTAGTGTGATTG	ACTCCAATTTGGCCCAAAAAAACCA
Streptococcus pneumoniae serotype 19A	CGG AAG GAA AAA TTC CAT TTG T	AAT GCG TCA ACC GTT TTG G	TCT TTT CGA CGA CGT ATC AGC TTC
Streptococcus pneumoniae serotype 19F	CCG AAA TTC TAA ATG AAT TGT ACT ACG A	GCA GTG AAA CAT TGG TGC CTA TAT	AAT TAA CTA GGC CCA TTT CCC CTA AT
Streptococcus pneumoniae serotype 20	AAA GAT ACT GGC TGA GGA GCT ATC TAT T	AGT CAA AAG TAC TCA ACC ATT CTG ATA TAT TC	AGG ATA AGG TCT ACT TTG TGG GAG TTC
Streptococcus pneumoniae serotype 21	GGTTTAAATATCGCTCCGGGTAT	CAAAAAAAGGGCTTGTAGACGAA	TGTGAATTGGACACGTTATGGAGC
Streptococcus pneumoniae serogroup 22A/F	TGA TGA ACA CTG GGT GGA TTG T	GCT TAT GGG CAC ATT CWC CAA	TTA AAT AAC CCA TTG GAA TTG AAA CGG
Streptococcus pneumoniae serotype 23A	GGGAATTGGCACTCTTCTGAAT	GATCGGCAAATGTTGAAACCA	TTGGCGGTAAACAATTAAGGCGT
Streptococcus pneumoniae serotype 23F	TGC TAT TTG CGA TCC TGT TCA T	AGA GCC TCC GTT GTT TCG TAA A	TTT CTC CGG CAT CAA ACG TTA AG
Streptococcus pneumoniae serotype 29	TTCGAGTTGTGCCGTTTTTACA	GGCGTACCCACCTCTAAAATTTT	AGGAGTACGCAGAGAAAAGACTAGGATTCAA
Streptococcus pneumoniae serotype 31	GATAGCATTACAGATGTCACTAAGGGATT	TTGGCGCAGAAAATAGAAACC	TCAAACCCCCCACGTAAAACCGC
Streptococcus pneumoniae serotype 33F	CGA GAG AGA ATA TGA GGG AAT TGT TA	TCT CAA TCC CCG CAT TTA CTG	AGG AAA ACT GTG GTC ACG GTT CG
Streptococcus pneumoniae serogroup 35B/D	ATG GGA TTC TTG GGC AGA AA	GCA TCC GTA CTT CGT TCT CCT T	CTT GGG TTG GAA ACG CCT TGA
Streptococcus pneumoniae serogroup 35F/37	CGAATTCGGAAAACAATGTGTTT	TATGCAATTTAGCTGCAAAAAATCC	TTGACATTTTTCCTCTAGATGGTTAT
Streptococcus pneumoniae serogroup 38/25A/F	ATT GTT TAC TGT CAT ACT CCT GTT GGA	TGT TCC TTG TTT TCG CAA TCC	TTG GTG GGA CGA CTG GCT TCT

Supplementary Table S2. Frequency of co-colonization according to testing by one or two genes.

Time interval	26 d-11 m	12-23 m	24-35 m	36-47 m	48-59 m	60-70 m	Total	χ^2 for trend *P*-value
**Number of children/samples**	**305**	**201**	**207**	**224**	**191**	**84**	**1212**	
**Colonized with ≥2 serotypes/ *lytA*-positive samples (n/N, %)**	10/94 (10.6)	21/70 (30)	19/95 (20)	30/104 (28.8)	30/87 (34.5)	10/33 (30.3)	120/483 (24.84)	**0.0008**
**Colonized with ≥2 serotypes/ *lytA*- & SP2020-positive samples (n/N, %)**	10/80 (12.5)	18/63 (28.57)	18/86 (20.93)	27/92 (29.35)	27/75 (36.0)	9/28 (32.14)	109/424 (25.71)	**0.0017**
